# Tumor-Associated Macrophages: Critical Players in Drug Resistance of Breast Cancer

**DOI:** 10.3389/fimmu.2021.799428

**Published:** 2021-12-17

**Authors:** Maoyu Xiao, Jun He, Liyang Yin, Xiguan Chen, Xuyu Zu, Yingying Shen

**Affiliations:** ^1^ Cancer Research Institute, The First Affiliated Hospital, Hengyang Medical School, University of South China, Hengyang, China; ^2^ Department of Spine Surgery, The Nanhua Affiliated Hospital, Hengyang Medical School, University of South China, Hengyang, China

**Keywords:** tumor-associated macrophages, drug resistance, breast cancer, drug therapy, targeting tumor-associated macrophages

## Abstract

Drug resistance is one of the most critical challenges in breast cancer (BC) treatment. The occurrence and development of drug resistance are closely related to the tumor immune microenvironment (TIME). Tumor-associated macrophages (TAMs), the most important immune cells in TIME, are essential for drug resistance in BC treatment. In this article, we summarize the effects of TAMs on the resistance of various drugs in endocrine therapy, chemotherapy, targeted therapy, and immunotherapy, and their underlying mechanisms. Based on the current overview of the key role of TAMs in drug resistance, we discuss the potential possibility for targeting TAMs to reduce drug resistance in BC treatment, By inhibiting the recruitment of TAMs, depleting the number of TAMs, regulating the polarization of TAMs and enhancing the phagocytosis of TAMs. Evidences in our review support it is important to develop novel therapeutic strategies to target TAMs in BC to overcome the treatment of resistance.

## 1 Introduction

Of all female cancers, BC is the most common cancer with the highest morbidity and mortality in women (1). Moreover, the incidence and mortality of BC are predicted to go on with rising globally (2, 3).

According to the distinction of ER, PR, HER2 level BC could be subtyped as Luminal A (ER+/PR+, HER2+), Luminal B (ER+/PR+, HER2-), HER2amp (ER-/PR-/HER2+), and Basal-like/triple-negative (ER-, PR-, HER2-) (4, 5). The morbidity and mortality of different molecular subtypes of BC differ (6). Distinct subtypes of BC are usually treated with different ways in clinical management (7). Drug therapy can be roughly divided into endocrine therapy, chemotherapy, targeted therapy, and immunotherapy (8). In clinical treatment, endocrine therapy (ET) is seen as the standard treatment for Luminal BC, chemotherapy is the preferred treatment for TNBC, targeted therapy is commonly used for HER2 positive BC treatment, and immunotherapy has been demonstrated to have the best therapeutic efficacy in TNBC, so it is most widely used in the treatment of TNBC.

**Figure 1:**
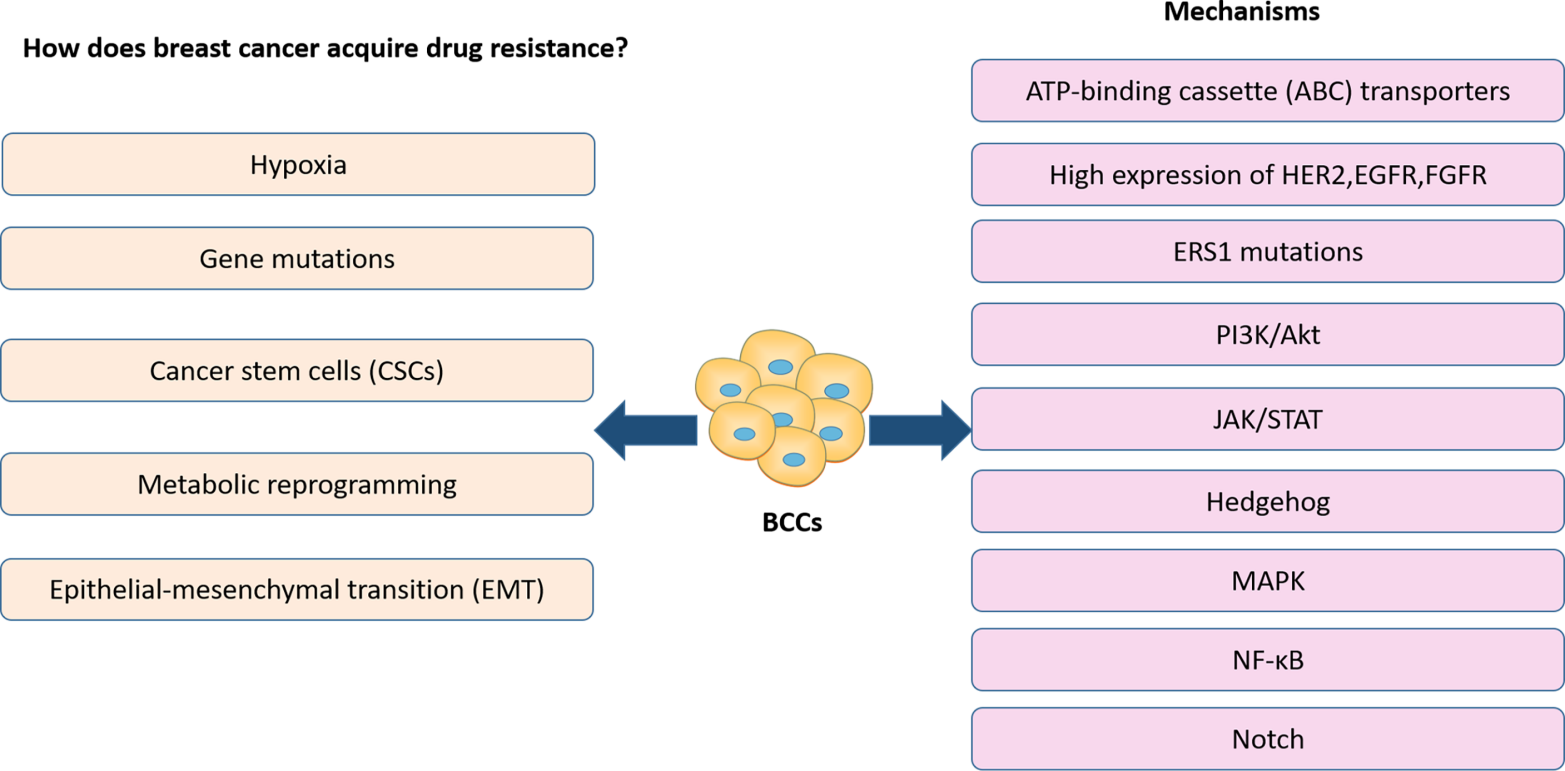
Previous reports on drug resistant in BC treatment.

Presently, the efficacy of treatments in BC has been limited by drug resistance (9), which has resulted in an increased focus on the study of drug resistance in BC. In the previous reports, the development of resistance to BC therapy is strongly linked to genetic mutations, metabolic reprogramming, breast cancer stem cells, EMT and hypoxia of the tumor microenvironment. These alterations are usually associated with the activation of a range of related signaling pathways, as well as the expression of associated signaling molecules (**Figure 1**). For example, high expression of HER2, EGFR, and FGFR is closely correlated with treatment resistance (10, 11). Activation of MAPK, PI3K-Akt signaling pathways also plays a critical role in treatment resistance of BC (12–16). Notch signaling pathway, Wnt signaling pathway activation, and ERS1 mutations are closely associated with breast cancer CSCs (17, 18). It has also been shown that breast cancer CSCs express elevated levels of ATP-binding cassette (ABC) transporters which pump drugs out of the cells (19). All signaling molecules and signaling pathways we mentioned above also play a significant role in the hypoxia and metabolic reprogramming of the tumor microenvironment (20). In addition, various signaling pathways, including NF-κB, Hedgehog, and JAK/STAT activation, affect BC treatment resistance by influencing the metabolic reprogramming of BCCs, CSC and EMT (21–26).

Recently, more and more evidence has revealed that one of the key mechanisms for drug resistance is the alteration of the tumor microenvironment (27–29). Macrophages are the most abundant immune cells in the tumor microenvironment (30, 31), and macrophages that reside in tumor microenvironment are referred to as TAMs (32). Classically, the macrophage phenotypes were divided into M1 and M2 macrophages. Macrophage phenotype is modulated by TIME (33–39). M1 phenotype exhibits proinflammatory responses and tumoricidal, whereas the M2 phenotype is anti-inflammatory and pro-tumor (40–42). Th1 cytokines such as interferon-γ (IFN-γ) or lipopolysaccharide (LPS) stimuli activate the M1 macrophages, and then promote the M1 macrophages secrete pro-inflammatory cytokines such as interlukin-6 (IL-6), interlukin-12 (IL-12), interlukin-23(IL-23), and tumor necrosis factors alpha (TNF-α). Comparatively, the M2 macrophage is enabled by Th2 cytokines such as IL-4, IL-13, glucocorticoids, and immunoglobulin complexes. M2 macrophages regulate angiogenesis, tissue remodeling, and wound healing (43–45). And most of the macrophages in the TIME are M2 macrophages (46, 47). M2 macrophages can encourage the growth, progression, angiogenesis and metastasis of various tumors such as nasopharyngeal, gastric and melanoma (48–50).

Therefore, TAMs are also considered as potential targets for reversing resistance of BC treatment. This review will focus on the current status of the treatment of various subtypes of BC, the impact of TAMs on the resistance of each subtype of BC, and an overview of the solutions to the resistance of BC.

## 2 The Role of TAMs on the Resistance of Breast Cancer Treatment

Many studies have demonstrated that TAM can induce tumor therapy resistance by enhancing CSC and involvement of the EMT (51), and TAM contributes to the development of therapy resistance in cancers by metabolic reprogramming to promote tumor angiogenesis (52). In addition, TAM secrets inflammatory cytokines and chemokines such as TNF-α, IL-6, CCL18 and so on to influence the therapeutic resistance of cancers (51, 53). As an important component of TIME, TAM induces immunotherapy resistance by inhibiting T-cell functions (54). It is noteworthy that the exosomal microRNAs (miRNAs) from cancer cells induces treatment resistance by regulating the polarization of the macrophage (53). In conclusion, TAM has a significant role in the development of treatment resistance in various tumors. Similarly, TAMs also play an important role in promoting drug resistance of different treatment options for BC. As shown in **Table 1** this section will focus on the impact of TAMs on resistance of various therapeutic agents, as well as the specific molecular mechanisms.

**Table 1:** The molecular mechanism of TAM-mediated BC treatment resistance.

Treatment Strategy	Drug Name	Signal Pathways	Cells/Tissues	TAMs responses	References
***Endocrine therapy***	Tamoxifen	PI3K/Akt/mTOR mTORC1-FOXK1	MCF-7 **THP-1**	Secret CCL2 Increase the recruitment	(55)
		HIF-1α/STAT3 EGFR/PI3K/Akt	T47D, MCF-7 **THP-1**	Polarize to M2 phenotype	(56)
		NF-Kb/STAT3/ERK	MCF-7	Secret TNF-α and IL-6	(57)
		NF-κB	**THP-1**	Form crown-like structures (CLS) Secret TNFα, IL-1β, IL-6 and PGE2	(58)
		EGFR overexpression	patients with postmenopausal, ER-positive, Her-2-negative, infiltrating ductal carcinoma	Increase the infiltration	(35)
	AIs	Jagged1-Notch	**THP-1**	Polarize to M2 phenotype	(59)
		Increased expression of CXCR4 signal	MCF-7	Increase the motility	(60)
	Others	Activation of c-Src, PKC and MAPK	MCF-7	Secret factors	(61)
		Akt/FOXO3a	TD47, MCF-7 MDA-MB-231	Secret TNF-α	(62)
***Chemotherapy***	Doxorubicin (Adriamycin)	NF-κB AP-1(c-jun)	RAW264.7 MCF-7	Secret IL-6	(63)
		PTEN/Akt	**THP-1**	Polarized to M2 phenotype	(64)
		IL-10R/STAT3/Bcl-2	BT-20, MDA-MB-453	Secret IL-10	(65)
	Paclitaxel	IL-10/STAT3/Bcl-2	T47D, BT549	Secret IL-10	(38)
		Unclear	**BMDM**	High expression of cathepsin	(66)
		insulin/IGF1R	4T1	Secret IGF	(67)
		MAPK/ERK kinase (MEK)	TS1	Secret factors	(68)
	Carboplatin	GJIC	T47D, MDA-MB-231 **PBMC, BMNC**	Direct cell–cell contacts	(69)
	Others	High expression of CSF-1	MCF-7	Increase the recruitment	(70)
		EGFR/STAT3/SOX2	4T07, 4T1	Secret EGFR	(71)
***Targeted therapy***	Olaparib	sterol regulatory element-binding protein 1 (SREBF1, SREBP1)	**PBMC, BMDM**	Glucose and lipid metabolic	(72)
	Lapatinib	Src/STAT3/ERK1/2-mediated EGFR signaling	SKBR3	Secret IL-8	(73)
	Trastuzumab	B7-H4 expression was upregulated	**PBMC, BMDM**	Immunosuppressive phenotype	(74)
	Bevacizumab	Fc-γ receptor and TLR4 High expression of IDO1	**RAW264.7** MDA-MB-231, 4T1	Polarize into M2b type	(75)
		Eotaxin and Oncostatin M.	MDA-MB-231, MCF-7	Polarize to M2 phenotype	(76)
	Vascular disrupting agents (VDAs)	CXCL12/CXCR4 axis	N202 **BMDM**	Increase the recruitment	(77)
	Hedgehog inhibitor -Cyclopamine	Hedgehog pathway	**THP-1**	M2 macrophages secret IL-6	(78)
	Ruxolitinib	JAK/STAT3	**THP-1**	Activation of JAK/STAT3	(79)
	BET inhibitor (JQ1)	IL-6 or IL-10/STAT3/IKBKE/NF-κB axis	MCF-7 MDA-MB-231 MDA-MB-468 BT549	Secret IL-6 and IL-10	(80)
	PI3K inhibitor- GDC-0941	NF-κB	4T1	Secret cytokines and chemokines	(81)
	Sorafenib	Unclear	**RAW264.7**	Increase the recruitment	(82)
***Immunotherapy***	CTLA-4 inhibitor	MSP-RON	**F4/80+ peritoneal macrophages**	High expression of PD-L1	(83)
	CD40 agonist	Unclear	**PMBC**	High expression of PD-L1	(84)
	Anti-PD-1 antibody	High expression of TYRO3	4T1	Reduce the ratio of M1/M2 TAMs	(85)
	Other	CD47-SIRPα	**Tumor mononuclear cells in 4T1 tumor-bearing mice**	High expression of SIRPα	(86)

### 2.1 Endocrine Therapy

**Figure 2:**
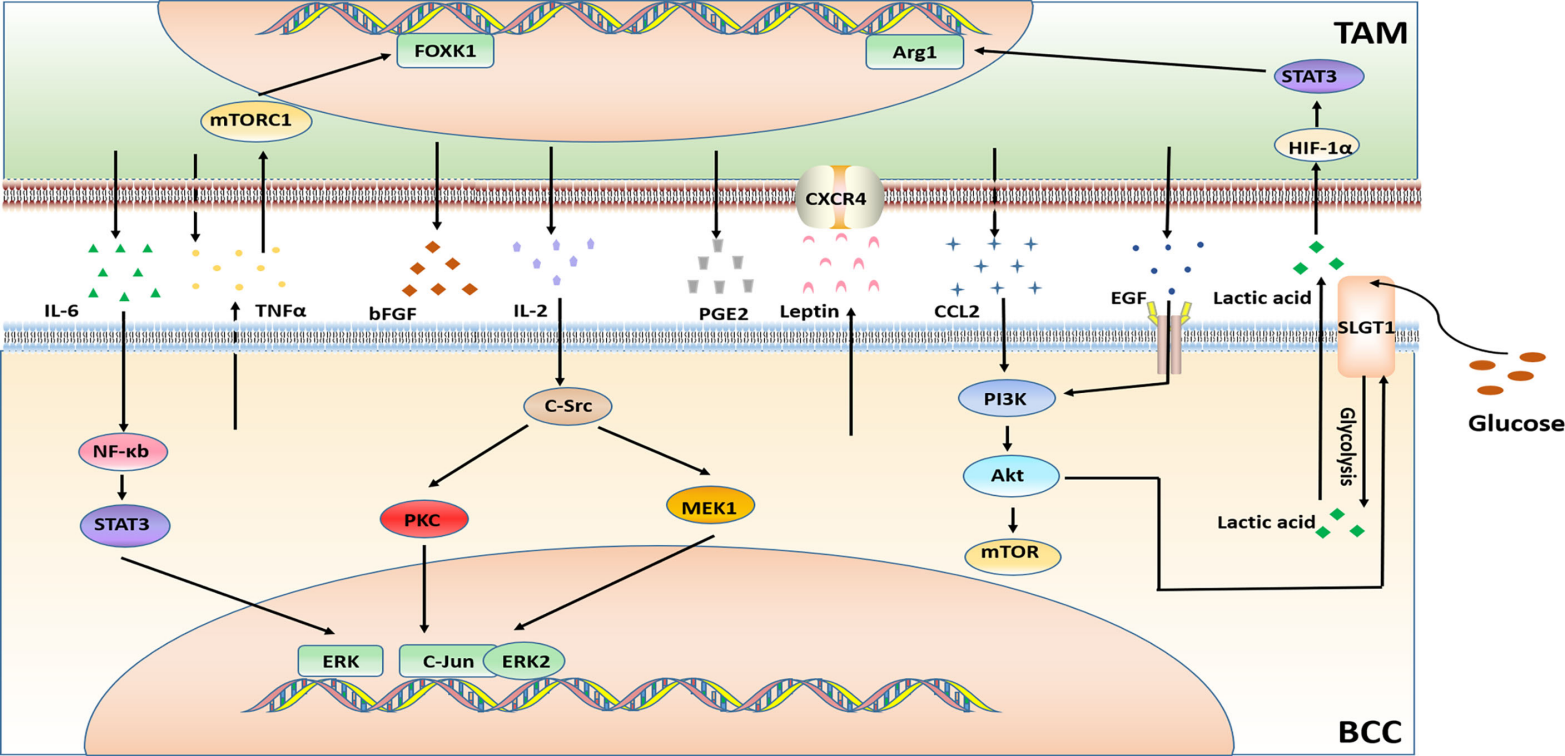
Major mechanisms of endocrine therapy resistance in BC in connection with TAMs.

Hormone receptor-positive (HR+), estrogen receptor-positive (ER+) and human epidermal growth factor receptor 2 negatives BC (i.e. Luminal BC) accounting for two-thirds of all BC cases, and endocrine therapy (ET) is the standard treatment for Luminal BC (87). According to the different mechanisms of endocrine therapy drugs, they can be fairly divided into the following categories:1. Selective estrogen receptor modulators (SERMs) represented by tamoxifen. 2. The selective estrogen receptor down-regulator (SERD) represented by fulvestrant. 3. Aromatase inhibitors (AIs) which can be divided into steroid drugs (letrozole and anastrozole) and non-steroid drugs (simestane and formestane) (88). 4. Ovarian function inhibitors represented by goserelin, also known as a gonadal hormone-releasing hormone antagonist (GnRha). This type of drug is rarely used in BC treatment alone and often combinated with other endocrine therapy drugs (89). Although these endocrine treatment programs have achieved satisfactory therapeutic effects clinically, there are still many patients who have *de novo* drug resistance or acquired drug resistance after long-term treatments. Therefore, it is necessary in order to investigate the mechanisms of resistance. And the possible mechanisms that may result in resistance to endocrine therapy in BC are given in **Figure 2**.

#### 2.1.1 Selective Estrogen Receptor Modulators (SERMs)- Tamoxifen

The cytokines generation in TAMs and breast cancer cells (BCCs) bring multiple signaling pathways crosstalk and then contribute to tamoxifen resistance. For instance, the secretion of CCL2 by TAMs activates the PI3K/Akt/mTOR signaling pathway in BCCs to increase the resistance of BCCs and the recruitment of TAMs, while drug-resistant BCCs secrete TNF-α, activate mTORC1-FOXK1in TAMs, and promote TAMs to polarize to M2 phenotype, secreting more CCL2 to form a positive feedback loop (55). And the glucose transporter SGLT1 is overexpressed in tamoxifen-resistant BCCs, which is expected to result in enhance glycolysis and lactic acid secretion. The BCCs-derived lactic acid promotes TAMs to polarize to M2-like *via* the HIF-1α/STAT3 pathway. Furthermore, the M2-like TAMs activate the EGFR/PI3K/Akt pathway and ultimately upregulate SGLT1 expression to promote tamoxifen resistance in ER-positive BCCs (56). Studies also have shown that TNF-α and IL-6 secreted by TAMs activate the NF-κB/STAT3/ERK signaling pathway in BCCs and the hyperphosphorylation of Erα, resulting in overexpression of Cyclin D1, c-Myc, CCL2 (MCP-1) and IL-6. Eventually, the tamoxifen resistant is further strengthened (57). Recently, multiple studies have revealed that TAMs in the tumor tissues of obese BC patients secretes cytokines which may also be related to tamoxifen resistance. The presence of necrotic adipocytes surrounded by macrophages forming crown-like structures (CLS) is associated with activation of NF-ĸB in macrophages and increase levels of TNFα, IL - 1β, IL-6, and PGE2 in TIME (58, 90, 91). In addition, tamoxifen resistance is related to high expression of EGFR, and the TAMs infiltration are also increased in BC tissues with high expression of EGFR, so the interaction between TAMs and BCCs with high expression of EGFR may also be the reason of tamoxifen resistance (35). Further studies are still needed to investigate the potential mechanism between TAMs and tamoxifen resistance in a large cohort.

#### 2.1.2 AIs

Research has implicated that Jagged1-Notch pathway in TAMs promotes AI resistance by polarizing TAMs to the M2 phenotype in BC directly (59). However, another mode of action is linked with the interplays between TAMs and BCCs. Anastrozole-Resistant (AnaR) BCCs release leptin, regulate the morphology of macrophages, and increase the motility of macrophages through the CXCR4 signaling. In turn, the activated macrophages promote the growth and movement of AnaR cells in the co-culture system. Consequently, leptin may play a crucial role in AI-resistant phenotype of TAMs in breast cancer (60). Furthermore, macrophages enhance aromatase activity and increase production of estrogen by synthesizing a variety of cytokines such as basic fibroblast growth factor (bFGF), interleukin 2 (IL-2), interleukin 6 (IL-6), tumor necrosis factor-α (TNF-α), prostaglandin 2 (PGE2) and so on (92–95). Although the specific molecular mechanism remains to be elucidated. These studies collectively outline the key role of TAMs in inducing AIs resistance in breast cancer.

#### 2.1.3 Others

In 2011, Stossi et al. also discovered additional molecular mechanisms that caused resistance of endocrine therapy in BC. Macrophage-derived factors through the hierarchical activation of multiple kinases (c-Src, PKC and MAPK) in BCC leads to the recruitment of the complex of ERK2 and c-jun to chromatin, which results in the transcriptional inhibition of ESR1 gene and the loss of ERα expression, thereby producing endocrine therapy resistance in BC (61). Besides, inflammatory macrophage-derived TNFα downregulates estrogen receptor α *via* activating the Akt pathway which phosphorylates FOXO3a in BCCs (62). This study also has suggested that TNF-α antagonists may reduce endocrine therapy resistance in BC.

Overall, the current research about the effect of TAMs on BC resistance to endocrine therapy mainly spotlights on the most common drugs in SERMs (tamoxifen) and aromatase inhibitors. The influence of TAMs on the resistance of other endocrine therapies is not clear, so further research is needed.

### 2.2 Chemotherapy

**Figure 3:**
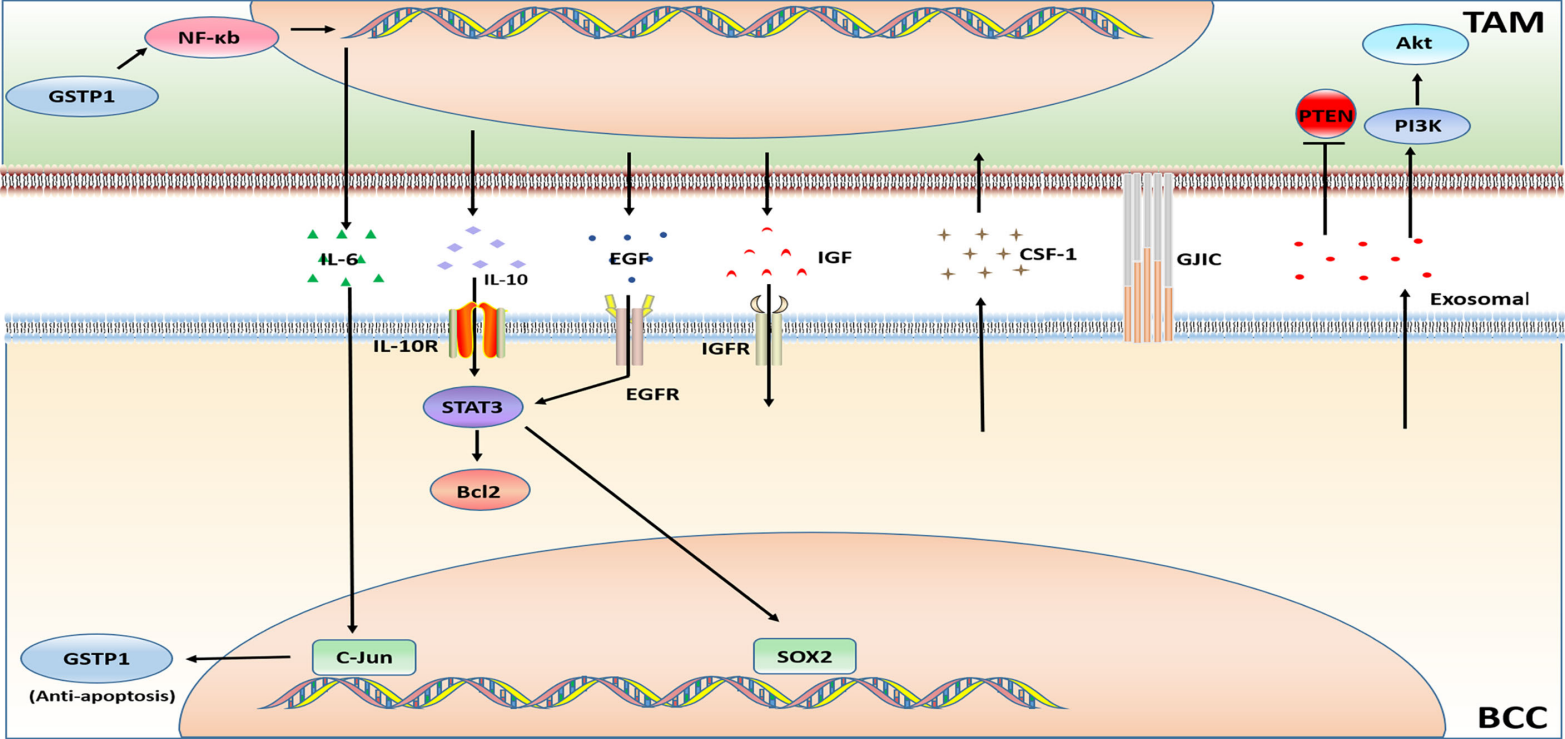
Major mechanisms of chemotherapy resistance in BC in connection with TAMs.

Triple-negative breast cancer (TNBC) is negative for ER, PR, and HER2 expression with a poor prognosis. Chemotherapy is the preferred treatment for this subtype of BC, but the benefits are not universal. The chemotherapeutic drugs are commonly used in BC treatment can be roughly divided into two categories, one type achieves anti-tumor effects by affecting tumor cell DNA synthesis or DNA base pairing, and the other is through interference with cell mitosis. The drugs that interfere with DNA synthesis includes anthracyclines, such as doxorubicin (adriamycin); antimetabolites, such as capecitabine, gemcitabine, methotrexate, and fluorouracil; platinum drugs such as carboplatin and cisplatin; and the alkylating agent cyclophosphamide. The drugs that interfere with tumor cell mitosis (i.e. microtubule inhibitors) include paclitaxel, docetaxel, etc. The chemotherapy resistance of BC is a serious challenge in the process of BC treatment. TIME is at the heart of the occurrence of drug resistance. Since TAMs are an important component of TIME, their influence on the chemotherapy resistance of BC cannot be ignored. The mechanism of TAM related to BC chemotherapy resistance is illustrated in **Figure 3**.

#### 2.2.1 Doxorubicin (Adriamycin)

The production of cytokines IL-6 and IL-10 causes doxorubicin resistance. Glutathione S-transferase P1 (GSTP1) is a phase II detoxification enzyme, and researchers has discovered that overexpression of GSTP1 notably activates the NF-κB signaling pathway in TAMs and increases the expression of IL-6, which further up-regulates GSTP1 in BCC through AP-1 (c-Jun), eventually leads to doxorubicin resistance (63). Similar to the above mentioned, drug-resistant BCCs and TAMs secrete IL-6 in a paracrine manner. IL-6 may increase the uptake of hemoglobin-binding globin complex by macrophages *via* stimulating the expression of CD163 in TAMs. Thereby increasing iron deposition in TAMs. The iron deposit further promotes the polarization of TAMs to the M2 phenotype, and then augments the secretion the secretion of IL-6, so that chemotherapy-sensitive BCCs also developed into doxorubicin resistance (96).

Unlike the above studies, a study has found that the breast tumor shed membrane vesicles called microparticles (MPs) which promote strong resistance to the doxorubicin. Drug-resistant BC derived MPs induce CD44- dependent clustering of TAMs, and induces pro-inflammatory cytokine secretion by TAMs, further stimulating the engulfment of TAMs by BCCs (97). The exosomes deliver microRNAs could influence TAMs. Doxorubicin-resistant BCCs secret exosomal miR-222 to directly target PTEN and cause Akt cascade activation and TAMs M2 polarization to induce tumor progression (64).

In addition, a study on TNBC resistance showed that TAMs-mediated doxorubicin resistance can be explained by TAMs secrete high levels of IL-10 and activation of IL-10/IL-10 receptor/STAT3/Bcl-2 signaling pathway (65).

#### 2.2.2 Paclitaxel

The factors generated by TAMs also affect paclitaxel resistance. M2-like TAMs in the tumor microenvironment secrete high levels of IL-10, and also induce drug resistance through the IL-10/STAT3/Bcl-2 signaling pathway (38). TAMs reduce the tumor cell death induced by paclitaxel, etoposide and doxorubicin through the high expression of cathepsin (including cathepsin B and cathepsin S) (66). Overexpression of insulin-like growth factors 1 and 2 (IGFs) in TAMs activates insulin/IGF1R signaling pathway in TNBC cells. Experimental results have suggested that insulin/IGF1 receptor activation and/or interstitial expression of IGF may be one of the principal reasons leading to paclitaxel resistance (67). Additionally, secretion factors of TAMs inhibit paclitaxel-induced mitotic arrest, DNA damage signals and cell death, leading to the occurrence of paclitaxel resistance (68).

Furthermore, a study on long non-coding RNAs (LncRNAs) reported that LINC00337 accelerated the malignant phenotype of BCCs and promoted chemoresistance to paclitaxel through M2-like macrophages (98).

#### 2.2.3 Carboplatin

Carboplatin resistance in BC treatment has also been shown to be related to M2 macrophages. The study verified a hypothesis that macrophages (MΦs) in the bone marrow stroma contribute to BCC dormancy. The dormant BCCs could behave like cancer stem cells (CSC). M2 type macrophages and CSCs form intercellular gap junction communication GJIC), causing carboplatin resistance of BCCs (69).

#### 2.2.4 Others

BCCs secrete CSF-1 to promote the recruitment of TAMs, which is also considered to be a cause of chemotherapy resistance. It was found that the use of a murinized, polyethylene glycol–linked antigen-binding fragment (Fab) against mouse (host) CSF-1 (anti-CSF-1Fab) in combination with the chemotherapy drugs cyclophosphamide, methotrexate and 5-fluorouracil reverse the chemotherapy resistance. The combination therapy reduces macrophage recruitment and downregulates the expression of chemoresistant genes and multi-drug resistance genes (70).

Cystathionine β-synthase (CBS) is an enzyme in the transsulfuration pathway. Overexpressed CBS in BCCs protected them from activated TAMs. CBS may significantly upregulate chemotherapeutic drugs resistance (99).

Thus far, substantial research efforts have revealed a closely correlation between TAMs and chemotherapy resistance. The development of BC chemotherapy resistance is connected with the polarization of TAMs, the generation of cytokines, the overexpression of drug resistance associated proteins, the activation of related signaling pathways and the recruitment of TAMs. These studies provide new targets and strategies for solving the chemotherapy resistance phenomenon in the clinical treatment of BC.

### 2.3 Targeted Therapy

**Figure 4:**
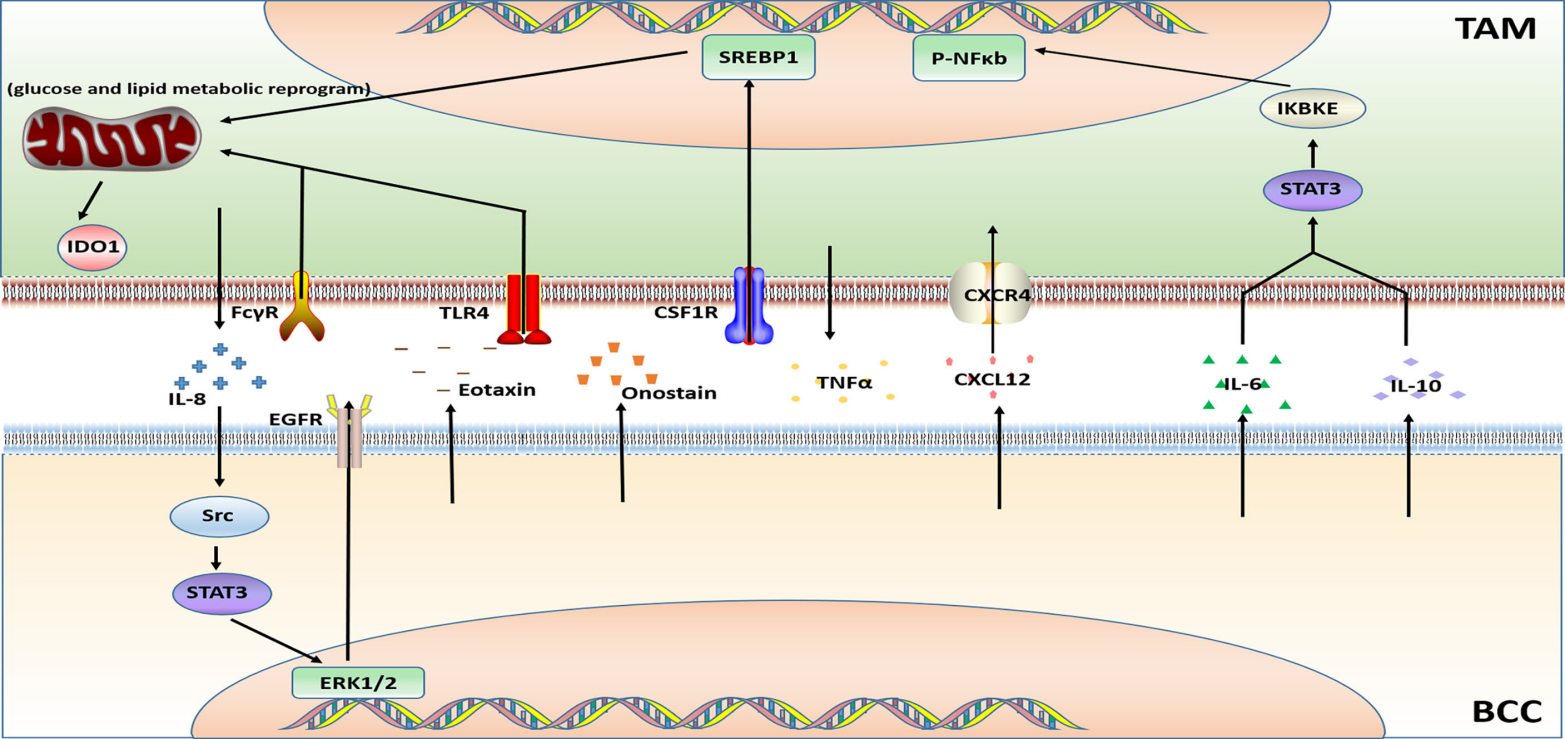
Major mechanisms of targeted therapy resistance in BC in connection with TAMs.

The therapeutic targets of breast cancer-targeted drugs currently approved by the Food and Drug Administration (FDA) are CDK4/6, mTOR, PARP, PIK3CA, TROP-2, PD-L1, HER2, and EGFR. However, there are only a few studies about the effect of TAMs on the resistance of these targeted drugs. This article will discuss the drugs and targets that have been reported like Olaparib, Lapatinib, Trastuzumab, Bevacizumab, and some novel targeted agents. **Figure 4** illustrates the relevant mechanism.

#### 2.3.1 Olaparib

PARP inhibitor Olaparib is a targeted drug for TNBC which governs the state, phenotype, function, and metabolism of TAMs. On the one hand, the recruitment of TAMs is increased, on the other hand, the PD-L1 and CSF1R are overexpressed. Moreover, Olaparib-treated TAMs functionally limit the proliferation and anti-tumor function of T cells. In summary, the data in the article published by Anita et al. indicated that through glucose and lipid metabolic rearrangement of TAMs driven by the sterol regulatory element-binding protein 1 (SREBF1, SREBP1) pathway after being treated with PARP inhibitors may limit the efficacy of PARP inhibitors and lead to the development of drug resistance (72).

#### 2.3.2 Lapatinib

Lapatinib is a small molecule tyrosine kinase inhibitor (TKI) of HER2 and VEGF. An article showed that the TAMs secrete IL-8 to activate Src/STAT3/ERK1/2-mediated EGFR signaling in BCCs and it participates in the resistance of HER2-positive BC to lapatinib (73).

#### 2.3.3 Trastuzumab

Trastuzumab, a humanized mAb utilized for HER2-overexpressing BC therapy. Bioinformatics analysis has revealed that TAMs as putative drivers of therapeutic resistance (100). Action modes of Trastuzumab include a) inhibition of HER2 mediated cell signaling (101), b) NK cell, T cell, mediated cytotoxicity (ADCC) and macrophage mediated phagocytosis (ADCP) (102, 103). However, TAMs undergoing ADCP after neoadjuvant treatment with trastuzumab have immunosuppressive phenotype and significantly upregulate B7-H4 in TAMs of HER2+ BC patients which cause the immune escape of BCCs. These effects on TAMs indicate that patients have a poor response after treatment with trastuzumab (74).

#### 2.3.4 Bevacizumab

Bevacizumab is a recombinant humanized monoclonal antibody that can bind VEGF to block the proliferation of endothelial cells and the formation of new blood vessels. Bevacizumab is always used in conjunction with chemotherapy drugs for recurrent or metastatic BC. TAMs are polarized into M2b type through Fc-γ receptor and TLR4 signaling pathway. The M2b type TAMs promote resistance to bevacizumab treatment by generating TNFα and activating the immunosuppressive factor IDO1 (75).

Similarly, a study published in 2014 also proved that hypoxia-induced BCCs secret Eotaxin and Oncostatin M, promoting the recruitment and polarization of macrophages into the M2 subtype. The polarization of TAMs into the M2 phenotype is an important reason for promoting breast tumor angiogenesis and leading to Bevacizumab resistance (76).

#### 2.3.5 Vascular Disrupting Agents (VDAs)

Poly (l-glutamic acid)-combretastatin A4 conjugate (PLG-CA4), a new type of Vascular disrupting agent (VDA). PLG-CA4 induces TAMs to polarize toward the M2-like phenotype, which induce the host’s immunosuppressive response, produce therapeutic resistance, and promote tumor growth (104).

In another study on VDA about combretastatin A4 phosphate (CA4P) found that CA4P-induced vascular stenosis, hypoxia, and hemorrhagic necrosis of mouse breast tumors were accompanied by the high level of chemokine CXCL12. Meanwhile, the number of proangiogenic TIE2-expressing macrophages (TEMs) is increased. Thus, the efficacy of CA4P was reduced and the tumor progression was accelerated (77).

#### 2.3.6 Hedgehog Inhibitor -Cyclopamine

M2-like TAMs were demonstrated to attenuate the efficacy of the Hedgehog (Hh) pathway inhibitor cyclopamine in BCCs. IL-6 derived from M2 macrophages mediates the resistance of BCCs to the hedgehog inhibitor Cyclopamine (78).

#### 2.3.7 JAK/STAT Inhibitor- Ruxolitinib

Previous studies have shown that signal transducer and activator of transcription (STAT) are important regulators of macrophage polarization (105). A study on targeted therapy resistance in TNBC uncovered that tumor-derived factors induced the activation of the JAK/STAT3 pathway in macrophages, leading to the BC resistance to JAK/STAT inhibitor ruxolitinib (79).

#### 2.3.8 BET Inhibitor (JQ1)

Previous studies have shown that BET protein controls cell growth, senescence, apoptosis, and differentiation (106, 107). BET protein also promotes tumor development by inducing the expression of some oncogenes such as C-MYC and FOXM1 (107). A study on the BET inhibitor indicated that TNBC-stimulated TAMs make TNBC cells resistant to BETi through the IL-6 or IL-10/STAT3/IKBKE/NF-κB axis (80).

#### 2.3.9 PI3K Inhibitor- GDC-0941

The drug resistance mechanism of GDC0941, an inhibitor against the target PIK3, was revealed in the study of Muhammad et al. *In vivo* ;experiments demonstrated that GDC-0941 inhibition of PI3K increased the infiltration of TAMs. The expression of macrophage-related cytokines and chemokines were induced at the same time. Also, by using the co-culture system *in vitro*, it was found that the presence of TAMs activated NF-κB signaling in BCCs, which made tumor cells resistant to PI3K inhibitors (81).

#### 2.3.10 Sorafenib

Sorafenib treatment cannot inhibit 4T1 tumor growth. However, the accumulation of TAMs and infiltration of M2-type TAMs was increased in 4T1 tumors after sorafenib treatment. Targeting TAMs inhibited the growth of 4T1 tumors. So TAMs played a prominent role in sorafenib resistance (82).

In addition to the commonly used targeted drugs, there are many new targeted drugs in the research and development stage. Among them, research on drugs targeting TAMs is of great significance to address the emergence of targeted treatment resistance in BC.

### 2.4 Immunotherapy

**Figure 5:**
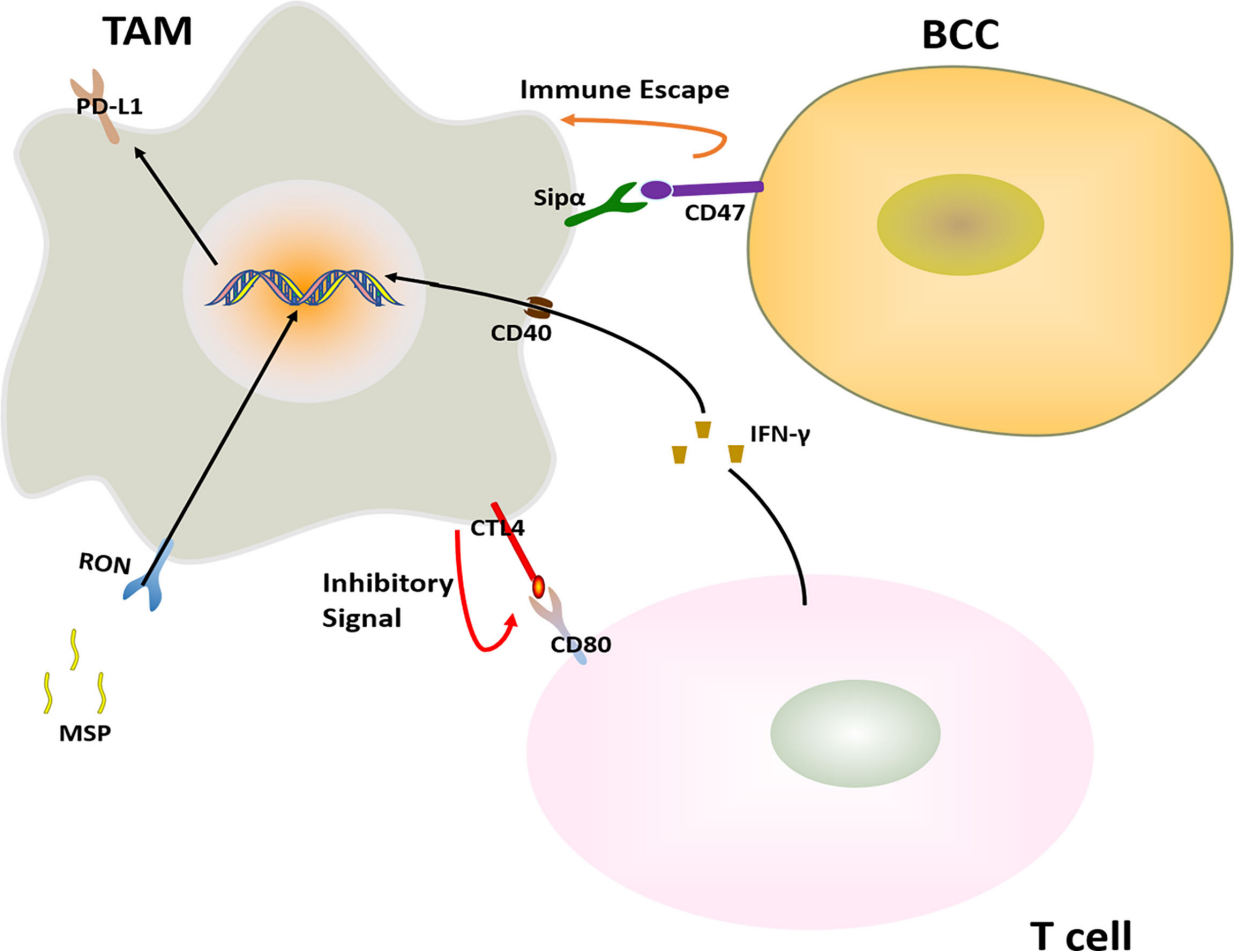
Major mechanisms of immunotherapy resistance in BC in connection with TAMs. The immunotherapy resistance is also associated with T cell.

Because the development of BC tumors is guided by the tumor microenvironment, immunotherapy is currently a hot spot in BC treatment research, especially for the treatment of refractory TNBC. Immunotherapy is a very promising and emerging strategy. At present, the most researched immunotherapies in clinical practice include chimeric antigen receptor (CAR) T-cell (CAR-T cell) immunotherapy, immunotherapy for NK cells, dendritic cells (DC), immune checkpoint inhibitors and so on (108). Although these immunotherapy programs have achieved good clinical therapeutic effects, the development of resistance is an inevitable problem. Studies have reported that TAMs were linked to immunotherapy resistance (109). In this section, the role of TAMs in the development of immunotherapy resistance is primarily discussed (**Figure 5**).

#### 2.4.1 CTLA-4 Inhibitor

The receptor tyrosine kinase RON expressed in macrophages inhibits the anti-tumor immune response. After RON was activated by its ligand MSP, the expression of PD-L1 was elevated in TAMs, and the MSP-RON signaling also up-regulates the binding of CD80 and CTLA-4 to inhibit T cell activation, reducing the effectiveness of immune checkpoint inhibitors in the treatment of BC (83).

#### 2.4.2 Agonistic Anti-CD40

Agonistic anti-CD40 treatment is a cancer immunotherapy that engagement of CD40 and agonistic antibodies. Agonistic anti-CD40 treatment gave rise to T cell activation. In parallel, T cell- and IFNγ-dependent upregulation of PD-L1 on TAMs. And this hinders the T cell response induced by anti-CD40, leading to BCCs immunotherapy resistance (84).

#### 2.4.3 Anti-PD-1 Treatment

BC tumors with high expression of TYRO3 inhibit tumor ferroptosis and support an immunosuppressive microenvironment by reducing the ratio of M1/M2 TAMs, thus promoting anti–PD-1 therapy resistance (85).

#### 2.4.4 Other

Commonly used immune checkpoint inhibitors in BC treatment include PD-1, PD-L1, and CTLA-4 inhibitors. Usually, the use of immune checkpoint inhibitors inhibits the development of BC well. However, due to the overexpression of the macrophage-suppressed CD47 signaling, and the presence of the CD47 receptor-inhibiting immune receptor signal regulator protein SIRPα on macrophages, the CD47-SIRPα pathway transmits “don’t eat me” to TAMs. The signaling helps tumor cells escape the elimination of the natural immune system (86).

Since immunotherapy is an emerging BC treatment strategy in recent years, many of the treatment options and drugs are still in the preclinical testing stage. In many other cancer immunotherapy-related studies, it has been shown that the resistance of immunotherapy and the improvement of therapeutic effects were significantly related to the interaction between TAMs and T cells, and the expression of PD-1/PD-L1 (110–112), but the related research of BC is still limited, thus the research on the immunotherapy resistance needs more exploration. Research on overcoming immunotherapy resistance by targeting TAMs has important clinical implications, and will greatly improve the efficacy of immunotherapy in BC.

In light of the above, the development of TAM-induced resistance to BC therapy can be broadly summarized as follows: 1. High expression of drug resistance-associated protein in TAM. 2. Activation of drug resistance-associated signaling pathways in BCC by TAM paracrine cytokines. 3. TAM exposure to cytokine stimulation from other cells in the tumor microenvironment, such as tumor cells, T cells and so on, which causes activation of relevant signaling pathways in TAM. Interestingly, these cells always interact with each other through a feedback loop but not act unilaterally, further increasing the drug resistance of BCCs.

## 3 How to Overcome Treatment Resistance in Breast Cancer by Targeting TAMs?

Because of the impact of TAMs on BC treatment resistance, various methods have been proposed to reduce or eliminate the resistance. As shown in **Table 2**, an abundance of studies has demonstrated that inhibiting the recruitment of TAMs, depleting the number of TAMs, regulating the polarization of TAMs and enhancing the phagocytosis of TAMs could be adopted as effective strategies to reverse drug resistance in BC treatment.

**Table 2:** Targeted TAMs to overcome therapeutic resistance strategies.

Target TAMs to overcome therapeutic resistance strategies	Concrete methods	References
***Inhibit the recruitment of TAMs***	Use TG100-115, the PI3Kγ selective inhibitor	(104)
	Interference with the CXCL12/CXCR4 axis	(77)
	The plasmid DNA encoding the CCL2 trap (pCCL2)	(113)
	Anti-cath-D antibody treatment	(114)
***Consume the number of TAMs***	Use the MAPK pathway inhibitors.	(68)
	Synthesize TAM-targeted probe (IRD-aCD206)	(82)
***Regulate the polarization of TAMs***	The agonist of STING receptors nanoparticle-incorporated cGAMP (cGAMP-NP)	(115)
	MCT-1 antagonists	(116)
	Block CSF1/CSF1R axis	(72)
	Photoimmunotherapy (PIT) using near-infrared-exposed Cetuximab-targeted gold nanorods (CTX-AuNR)	(65)
	Intratumoral injection of IL-21	(117)
	EED-IFNγ, an scFv protein containing an engineered effector domain (EED).	(118)
	Z-GP-DA VLBH through GM-CSF to induce repolarization of TAMs to the M1phenotype.	(69, 119)
	Activation of Toll-like receptor 4 (TLR4) Activation of NF-кB.	(119)
	Furin-responsive aggregated drug delivery system AuNPs-D&H-R&C	
	PC1/3-knockdown	(120)
***Enhance the phagocytic ability of TAMs***	Cetuximab-treated and cyclophosphamide treated (CTX-treated) combined with anti-EGFR therapy.	(121)
	Inhibit B7-H4	(74)
	Metformin induces miR‐708‐mediated suppression of CD47	(122)
***Synergistic influence of multiple mechanisms***	Block Eotaxin/Oncostatin M	(76)
	Construct a stimulus-responsive multifunctional nano-platform (ZIF-PQ-PDA-AUN)	(86)
	Use HA-coated PEiPLGA-MTX nanoparticles (NPs)	(79)
	CD47 blockade combined with Cowpea Mosaic Virus Nanoparticle (CPMV)	(123)
***Others***	Use the tea nanoparticles (TNPs)	(124)
	Use the AXL inhibitors	(125)

### 3.1 Inhibit the Recruitment of TAMs

As mentioned above, VDAs such as PLG-CA4 and CAP4 were capable of causing resistance to BC treatment. But it has been turned out the resistance can be reversed by reducing the recruitment of TAMs. For example, TG100-115, the PI3Kγ selective inhibitor, functions by reducing the recruitment of TAMs thereby significantly preventing tumor development and prolonging survival (104). As for CA4P, pharmacological interference with the CXCL12/CXCR4 axis can inhibit the recruitment of TAMs in CA4P-treated tumors, and dramatically improved the efficacy of CA4P treatment (77). Therefore, the combination of VDAs with drugs that inhibit the recruitment of TAMs will further improve the efficacy.

It is well known that CCL2 identified as cytokine intimately linked to tumor immunosuppressive microenvironment. Thus, the researchers designed a protein trap that binds with CCL2 with high affinity and specificity. Plasmid DNA encoding the CCL2 trap (pCCL2) is specifically delivered to the TIME by using targeted lipid-protamine-DNA (LPD) nanoparticles. The pCCL2 trap treatment successfully reduced the numbers of immunosuppressed M2-like TAMs. Hence, the pCCL2 treatment facilitated the checkpoint blockade therapy (113).

Human cathepsin D (cath-D) is produced by human epithelial BCCs, and high cath-D levels are associated with poor prognosis in human BC. *In vivo *study has revealed that anti-cath-D antibody-based treatment prevented the recruitment of TAMs which may be the important reason for limiting tumor growth (114).

### 3.2 Depletion the Number of TAMs

Depletion the number of the TAMs has also proved to be an important strategy to reverse BC treatment resistance. Inhibitors targeting the MAPK pathway have been shown to promote acute depletion of major histocompatibility complex class II low-molecular-weight (MHCIIlo) TAMs, increase paclitaxel-induced DNA damage and apoptosis in BCCs, so as to obtain a better curative effect (68).

As previously described sorafenib resistance has a relationship with M2-type TAMs. Researchers synthesized a TAM-targeted probe (IRD-aCD206) by conjugating a monoclonal anti-CD206 antibody with a near-infrared phthalocyanine dye to decrease the number of M2-type TAMs in breast tumors with the sorafenib resistance. IRD-aCD206 PIT inhibited the growth of 4T1 tumors and prevented metastasis to the lungs (82).

### 3.3 Regulate the Polarization of TAMs

In recent years, nanocomposites and photoimmunotherapy have become a hotspot in BC treatment. They can transform M2 type TAMs into M1 type TAMs, which may help reverse the “cold” tumor immune niche transition to “hot” tumor immune niche and resume immunotherapy based on immune checkpoint blockade (ICB) (126), thereby enhancing the effect of immunotherapy and reversing tumor resistance. The Stimulator of IFN genes (STING) is an exciting target for therapeutic adjuvants in the treatment of TNBC. A study in an agonist of STING receptors ‘cGAMP’ showed that nanoparticle-incorporated cGAMP (cGAMP-NP) stimulates STING more effectively by guiding TAMs to reprogram from M2-like phenotype to M1-like phenotype and enhancing anti-tumor immunity. Most importantly, these data has indicated that the system composed of cGAMP-NP regulated the TIME sufficiently and controlled PD-L1–insensitive TNBC (115). Additionally, the MCT-1/miR-34a/IL-6/IL-6R signal axis promotes TNBC Epithelial-mesenchymal transition (EMT) progression, tumor stem cell differentiation and M2 polarization. Conversely, MCT-1 antagonists inhibit the expression of IL-6R, TAMs are polarized toward M1, and induce the expression of the tumor suppressor gene miR-34a, thereby prevent the PD-1/PD-L1 interaction that increases anti-tumor activity and improving the efficacy of TNBC therapy (116).

Converting TAMs from M2 type to M1 type can not only enhance the efficacy of immunotherapy, but also have a positive impact on targeted therapy and chemotherapy resistance. PARP inhibitor treatment activates the CSF-1/CSF1R axis between TNBC cells and TAMs, thereby promoting immunosuppressive TIME. Reciprocally, blocking CSF1/CSF1R axis decreases the ratio of M2-like TAMs, and overcomes the resistance of PARP inhibitors (72). In 2021, a study on TNBC photoimmunotherapy (PIT) showed that PIT using near-infrared-exposed Cetuximab-targeted gold nanorods (CTX-AuNR) is a promising treatment strategy for treating EGFR-overexpressing TNBC cells, reversing tumor resistance, and regulating TIME (65). A study by Meng Xu et al. also proved that intratumoral injection of IL-21 enhanced tumor-specific CD8+ T cell anti-tumor response by transforming tumor-associated macrophages from M2 phenotype to M1 phenotype, thereby overcoming anti-Her2 resistance (117). EED-IFNγ, a scFv protein containing an engineered effector domain (EED). Treatment with EED-IFNγ increased the proportion of M1-type TAMs in breast tumors, and it is effective on tumors that are resistant to anti-HER2/neu antibody therapy (118). Among targeted therapies, the most interestingly, Z-GP-DA VLBH reverses multidrug resistance in BC, unlike other VDAs. It acts through GM-CSF to induce repolarization of TAMs to the M1phenotype (119).

Studies have shown that M2a-type TAMs activated by Toll-like receptor 4 (TLR4) were converted to M1 phenotype. M1 phenotype TAMs reverse BCCs dormancy through activating NF-кB. It sensitizes BCCs to carboplatin and improves the survival rate (69). Moreover, scientists designed a furin-responsive aggregated drug delivery system AuNPs-D&H-R&C to overcome chemoresistance by reprogramming M2-like TAMs to anti-tumor M1 phenotype (127).

The proprotein convertase 1/3(PC1/3) expressed in TAMs regulated immune response as the Toll-like receptor. PC1/3-knockdown promotes TAMs toward M1 phenotype, and TAMs secreted factors attracted naïve T helper lymphocytes to promote the cytotoxic response and further decrease the resistance of BCCs (120).

However, studies on the use of adjuvant/sensitizers/combination therapy/plant-derived drugs to modulate the polarization of TAMs in BC to overcome treatment resistance are scarce, but intervention of TAMs polarization by pharmacotherapy to overcome resistance has been investigated in other tumors. For example, in cisplatin-resistant lung cancer, dasatinib treatment reverses the polarization of TAMs to the M1 type, thereby improving the sensitivity of lung cancer to cisplatin (128). Likewise, triptolide (TPL) enhances the therapeutic effect drug-resistant ovarian cancer to chemotherapeutic agents by reversing the polarization of M2 macrophages (129). Similar findings have been issued in the studies on pancreatic ductal adenocarcinoma (PDAC) and head and neck squamous cell carcinoma (130, 131). These results allow us to hypothesize that using different types of drugs or combination therapy to target regulating polarization of TAMs in BC may reverse the drug resistance. And this may become a new research hotspot in the future to address the treatment resistance status of BC.

### 3.4 Enhance the Phagocytic Ability of TAMs

In the strategy of targeting TAMs to resist BC treatment resistance, improving the phagocytic function of TAMs is also a very effective solution. Bone marrow metastasis of BC is usually related to treatment resistance (132). Experiments have shown that in untreated, cetuximab-treated and cyclophosphamide treated (CTX-treated) mice, the bone marrow is full of tumor cells, while in CTX plus cetuximab-treated mice, most of the tumor cells in the bone marrow are cleared. These results indicated that CTX and anti-EGFR therapeutic drugs have a synergistic effect to eliminate BCCs in the bone marrow. CTX enhances the phagocytic function of macrophages and overcomes drug resistance by up-regulating activation of FcgRs (121).

Observation has proved that trastuzumab could lead to treatment resistance. And B7-H4, a key immune checkpoint molecule in TAMs, plays a major role in the resistance. Trastuzumab treatment increases the expression of B7-H4 in TAMs, and the anti-tumor function of NK cells and tumor-specific T cells was inhibited. While the B7-H4 is inhibited, the phagocytic function of TAMs is enhanced, so the therapeutic effect of trastuzumab on HER2+ BC is significantly enhanced (74).

Previous studies showed that miR‐708 as a potential tumor suppressor. Metformin attenuates the breast cancer stem cells (BCSCs) through miR‐708‐mediated suppression of CD47, and promotes phagocytosis of BCSCs by TAMs, increasing BCCs sensitivity to chemotherapeutic agents (122).

### 3.5 Synergistic Influence of Multiple Mechanisms

In fact, TAMs often undergo various alterations simultaneously to improve the sensitivity of BC treatment.

For example, blocking Eotaxin/Oncostatin M not only hinders the recruitment of TAMs but also prevents them from differentiating into tumor-friendly M2 macrophage subgroups, thereby enhancing the anti-angiogenic effect of Bevacizumab and minimizing the possibility of drug resistance due to the promotion of angiogenesis mediated by M2-like TAMs (76).

Compared with the early single immunotherapy, nanocomposite materials show stronger anti-tumor immunity in BC. A study has constructed a stimulus-responsive multifunctional nano-platform (ZIF-PQ-PDA-AUN) promoted T lymphocytes enter the tumor site, enhanced the phagocytic function of TAMs and significantly reversed the pro-tumor M2-like TAMs to anti-tumor M1-like TAMs (86).

Another study has found that naked or HA-coated PEiPLGA-MTX nanoparticles (NPs) used alone or in combination with PD-L1 antibody promoted the immune regulation of BC tumor microenvironment and significantly reduce primary tumors and metastasis. This study reported that M2 TAMs involved in NPs-mediated inhibition of the IL-10/STAT3/NF-κB signal axis, thereby mediate the regulation of the survival genes, the drug-resistant genes apoptosis-related genes and reducing drug resistance ultimately. In addition, the expression of immunosuppressive factor (IL-10, TGF-β, and PD-L1) is reduced, transforming TAMs from M2 type to M1 type, so the anti-tumor cytotoxic immune response is enhanced (133).

Similarly, in a study on CD47 blockade and Cowpea Mosaic Virus Nanoparticle (CPMV), the authors found that CD47 blockade combined with *in situ* CPMV vaccine in the 4T1 breast tumor model showed synergistic anti-tumor activity. The researchers observed that CPMV treatment increased the recruitment of CD86+ and major histocompatibility complex class II (MHCII) highly-expressed TAMs, thus significantly enhanced phagocytic capacity of TAMs, ultimately resulting in a powerful anti-tumor immune response (123).

### 3.6 Others

And researchers have developed an infusion-dialysis procedure for isolating tea nanoparticles (TNPs) that enhance the secretion of cytokines as well as the chemokines from TAMs. More importantly, the DOX-loaded TNPs markedly increased the DOX uptake in MCF-7/ADR multidrug resistant BCCs (124). So, this evidence indicated that the TNPs may exert an antitumor effect *via* modulating the function of TAMs immune.

AXL inhibitors reduce HIF-1α levels and alter the hypoxic response, leading to the production of key cytokines for TAMs. The observation suggests that inhibition of Axl generates a setting to increase immunotherapy. Accordingly, combining Axl inhibitors with anti–PD-1 in HER2+ BC reduces the primary tumor and metastatic burdens (125).

Overall, the development of nanomaterials, inhibiting key signaling axes, and using of antibodies to related proteins could modulate the function of TAMs as well as alter the number of TAMs. Targeting TAMs has become an important strategy to solve the resistance of BC treatments.

## 4 Conclusion and Perspectives

In summary, TAMs, as an indispensable part of the tumor microenvironment of BC, have an important impact on the occurrence of BC treatment resistance. TAMs promote the development of BC treatment resistance not only based on the unilateral effect of TAM on BCCs but through a feedback loop interaction between TAMs and BCCs in many cases. In addition to the conventional pharmacological approaches mentioned earlier, TAMs have also been found to play an important role in resistance to some new approaches of treating BC (134). Therefore, targeting TAMs to overcome resistance to BC treatments and enhance the efficacy of drug therapy has become a promising strategy. Regulation of TAMs polarization may be the most promising therapeutic modality compared to the reduction of TAMs recruitment as well as depletion of the number of TAMs. Through the regulation of TAMs polarization, the function of TAMs and the distribution of relevant cytokines in the tumor microenvironment can be affected, thus regulating BC treatment resistance. It is also worth mentioning that the interconnection of the biophysical tumor microenvironment can also influence tumor progression by regulating the polarization of TAMs. BCCs are able to acidify the extracellular environment, and the higher the degree of malignancy, the stronger the ability to acidify the tumor microenvironment (135). Tumor acidosis induces polarization of TAMs to the M2 type in a variety of cancers, including BC (136–138). In addition, proton pump inhibitors can reverse H (+) ion homeostasis in the tumor microenvironment and induce tumor cell death (139). Some *in vivo* and *in vitro* experiments have shown proton pump inhibitors enhance the anti-tumor effect of TAM, but their exact role in BC needs to be further studied (139–141). Therefore, reducing the occurrence of resistance in BC by focusing on the alteration of the biophysical tumor microenvironment to regulate the polarization of TAMs may also be a promising therapeutic strategy in the future. At present, scientists have built new platforms and biophysical models that are different from traditional models of tumor microenvironment. For instance, Xuefei Li et al. investigated the crosstalk between cancer cells and macrophages in the tumor microenvironment based on silicon (Computational) co-culture model. The interaction between TAM polarization and epithelial-mesenchymal epithelial of cancer cells was studied (142). In addition, an in silico model have been developed to study the effect of TAM on tumor cell migration (143). Through these new models, we can more accurately study the influence of various components in TIME on tumor therapy, which is also conducive to the study of targeting TAM to overcome drug resistance in BCC treatment (144–146). So, the knowledge of BC immunotherapy needs further investigation in the future. In addition, TAMs can interact with other immune cells in the tumor microenvironment, such as T cells, DC cells, NK cells, to play breast tumors in the development, metastasis, and treatment resistance significant influence. Therefore, exploring various immune cells and their crosstalk in the immune microenvironment may also play a critical role in the development of drug therapy for refractory BC. In conclusion, we speculate that the combination of targeted TAM with existing BC treatments will be an important strategy to overcome BC drug resistance.

## Author Contributions

MX and JH conceived and drafted the manuscript. LY discussed the concepts of the manuscript. XC helped drew the figures. YS and XZ approved the version to be submitted.

## Funding

This work was supported by the Project of National Natural Science Foundation of China (81972487, 81502276), Project of Natural Science Foundation of Hunan Province (2021JJ20039, 2020JJ4551), Project of Health Commission of Hunan Province (20201926, 202104070680).

## Conflict of Interest

The authors declare that the research was conducted in the absence of any commercial or financial relationships that could be construed as a potential conflict of interest.

## Publisher’s Note

All claims expressed in this article are solely those of the authors and do not necessarily represent those of their affiliated organizations, or those of the publisher, the editors and the reviewers. Any product that may be evaluated in this article, or claim that may be made by its manufacturer, is not guaranteed or endorsed by the publisher.
